# QcrC is a potential target for antibody therapy and vaccination to control *Campylobacter jejuni* infection by suppressing its energy metabolism

**DOI:** 10.3389/fmicb.2024.1415893

**Published:** 2024-07-02

**Authors:** Koji Hosomi, Noritoshi Hatanaka, Atsushi Hinenoya, Jun Adachi, Yoko Tojima, Mari Furuta, Keita Uchiyama, Makiko Morita, Takahiro Nagatake, Azusa Saika, Soichiro Kawai, Ken Yoshii, Saki Kondo, Shinji Yamasaki, Jun Kunisawa

**Affiliations:** ^1^Laboratory of Vaccine Materials, Microbial Research Center for Health and Medicine, National Institutes of Biomedical Innovation, Health and Nutrition (NIBIOHN), Osaka, Japan; ^2^Laboratory of Gut Environmental System, Microbial Research Center for Health and Medicine, National Institutes of Biomedical Innovation, Health and Nutrition (NIBIOHN), Osaka, Japan; ^3^Graduate School of Veterinary Science, Osaka Metropolitan University, Osaka, Japan; ^4^Asian Health Science Research Institute, Osaka Metropolitan University, Osaka, Japan; ^5^Osaka International Research Center for Infectious Diseases, Osaka Metropolitan University, Osaka, Japan; ^6^Laboratory of Proteomics for Drug Discovery, NIBIOHN, Osaka, Japan; ^7^Laboratory of Functional Anatomy, Department of Life Sciences, School of Agriculture, Meiji University, Kawasaki, Japan; ^8^Institute of Molecular and Cell Biology, Agency for Science, Technology and Research, Singapore, Singapore; ^9^Graduate School of Medicine, Osaka University, Osaka, Japan; ^10^Graduate School of Pharmaceutical Sciences, Osaka University, Osaka, Japan; ^11^Graduate School of Dentistry, Osaka University, Osaka, Japan; ^12^Graduate School of Science, Osaka University, Osaka, Japan; ^13^International Vaccine Design Center, The Institute of Medical Science, The University of Tokyo, Tokyo, Japan; ^14^Department of Microbiology and Immunology, Kobe University Graduate School of Medicine, Kobe, Japan; ^15^Research Organization for Nano and Life Innovation, Waseda University, Tokyo, Japan

**Keywords:** enteric infectious disease, *Campylobacter*, inflammation, bacterial metabolism, cytochrome, vaccine, antibody

## Abstract

**Introduction:**

*Campylobacter* spp. are a public health concern, yet there is still no effective vaccine or medicine available.

**Methods:**

Here, we developed a *Campylobacter jejuni*-specific antibody and found that it targeted a menaquinol cytochrome *c* reductase complex QcrC.

**Results:**

The antibody was specifically reactive to multiple *C. jejuni* strains including clinical isolates from patients with acute enteritis and was found to inhibit the energy metabolism and growth of *C. jejuni*. Different culture conditions produced different expression levels of QcrC in *C. jejuni*, and these levels were closely related not only to the energy metabolism of *C. jejuni* but also its pathogenicity. Furthermore, immunization of mice with recombinant QcrC induced protective immunity against *C. jejuni* infection.

**Discussion:**

Taken together, our present findings highlight a possible antibody- or vaccination-based strategy to prevent or control *Campylobacter* infection by targeting the QcrC-mediated metabolic pathway.

## Introduction

Enteric bacterial infections are a global public health concern because of their high mortality and morbidity, particularly among children in developing countries (GBD Diarrhoeal Diseases Collaborators, 2017). Their threat is increasing in concert with a rise in antibiotic resistance; therefore, new antimicrobial treatments are urgently needed.

*Campylobacter* infection is a leading cause of diarrheal mortality in children younger than 5 years, accounting for approximately 30,000 deaths worldwide in 2015 (GBD Diarrhoeal Diseases Collaborators, 2017). Even in developed countries such as the United States of America and Japan, *Campylobacter* infections are common with 9,723 and 1995 total cases reported, respectively, in 2018 (Tack et al., 2019; Vetchapitak and Misawa, 2019). Furthermore, molecular mimicry between the sialylated lipooligosaccharide structures of *Campylobacter* and ganglioside epitopes on human nerves leads to cross-reactive immune responses that are thought to trigger severe neuronal disorders such as Guillain–Barré syndrome and Miller Fisher syndrome (Nyati and Nyati, 2013; Hameed et al., 2020). Therefore, *Campylobacter* infection is not only a short-term risk as a foodborne illness, but it may also result in conditions that necessitate much longer-term care.

*Campylobacter jejuni* is a gram-negative, microaerophilic, spiral-shaped bacterium, and this organism most commonly causes inflammatory diarrhea, fever, cramps, and nausea in humans (Platts-Mills and Kosek, 2014; Burnham and Hendrixson, 2018). To prevent *Campylobacter* infections, several vaccine candidates have been developed up to the clinical stage but ultimately they have all been unsuccessful (Poly et al., 2019; Frost et al., 2023). For example, a *C. jejuni* whole-cell vaccine derived from *C. jejuni* strain 81–176 was trialed in humans but it was abandoned due to a high risk of inducing neuronal disorders (Poly et al., 2019). Then, *C. jejuni* vaccinology efforts switched to an approach using component vaccine antigens such as flagellin or the capsule (Poly et al., 2019; Frost et al., 2023). However, there are currently no vaccines under active clinical development that target *C. jejuni* (Frost et al., 2023). Thus, *Campylobacter* infections continue to be an issue that threatens long-term health at the global scale.

Here, we established monoclonal antibodies by means of whole-cell immunization (Kurashima et al., 2012) and found a clone that specifically targeted a functional molecule that is conserved across multiple *C. jejuni* strains, including several clinical strains, but is not expressed by related species such as *C. coli* and *C. fetus*. This functional molecule was ultimately identified as a menaquinol cytochrome *c* reductase complex QcrC, the expression of which is associated with the pathogenicity of *C. jejuni*. This approach allowed us to simultaneously identify both a *C. jejuni*-targeting antibody and a novel antigen for further development as strategies for the prevention, diagnosis, and treatment of *C. jejuni* infection.

## Methods

### Study approval

Animal experiments were approved by the Animal Care and Use Committees of the National Institutes of Biomedical Innovation, Health and Nutrition (Approval No. DS27-48R10) and of Osaka Metropolitan University (Approval Nos. BS22-Shi-73 and BS22-Jitsu-89), and were conducted in accordance with their guidelines.

### Bacterial culture

*Campylobacter jejuni* strain 81–176 was cultured slightly aerobically at 37°C by using the AnaeroPack system (Mitsubishi Gas Chemical, Tokyo, Japan). The AnaeroPack system maintains an environment of 6–12% oxygen and 5–8% carbon dioxide. Columbia 5% Horse Blood agar plates (bioMérieux, Tokyo, Japan), Bolton broth (Oxoid), Bolton broth supplemented with 1.5% agar (Bolton agar plate) were used for the experiments.

In addition to strain 81–176, other *C. jejuni* strains, *C. coli*, *C. fetus*, *C. hyointestinalis*, *C. helveticus*, *Arcobacter butzleri*, *A. cryaerophilus*, and *Helicobacter pylori* were used for flow cytometry analysis to examine the reactivity of 2B1 mAb (Supplementary Table S3). These bacterial strains were cultured slightly aerobically at 37°C by using the AnaeroPack system and Columbia 5% Horse Blood agar plates.

For preparing a fecal bacterial cocktail, mouse feces (2–3 grains) were cultured anaerobically in 500 mL of brain heart infusion medium (BD Biosciences) at 37°C for 3 days, and bacterial cells were collected by centrifugation at 10,000 × g for 10 min at room temperature (25°C) and used for ELISA screening for *C. jejuni*-specific mAb clones.

### Development of hybridomas and screening for *Campylobacter jejuni*-specific clones

Female BALB/c mice (age, 7 weeks) were purchased from CLEA Japan, Inc. (Tokyo, Japan). *C. jejuni* strain 81–176 was heat-killed by incubation at 70°C for 30 min. The heat-killed *C. jejuni* was suspended in phosphate-buffered saline (PBS) at the concentration of 2 × 10^9^ CFU/mL and the cell suspension were mixed 1:1 with Sigma Adjuvant System (Sigma). Mice were immunized once by footpad injection with 100 μL of the mixture (10^8^ CFU/mouse). Popliteal and inguinal lymph nodes were collected 14 weeks after the injection and cell fusion using SP2/0 myeloma cells was performed as previously described (Kurashima et al., 2012). Culture supernatants were tested for *C. jejuni*-specific antibodies 7 days after fusion by ELISA, as previously described (Hosomi et al., 2019). In brief, 96-well immunoplates (Thermo Fisher Scientific) were coated with 100 μL of lyophilized heat-killed *C. jejuni* (1 mg/mL) or fecal bacterial cocktail (1 mg/mL) diluted with PBS at 4°C overnight. The plates were blocked with 1% (w/v) bovine serum albumin in PBS, the culture supernatants were added to the wells and the plates were incubated for 2 h at room temperature (25°C). The plates were then incubated with goat anti-mouse IgG conjugated with horseradish peroxidase (Southern Biotech) and tetramethylbenzidine peroxidase substrate, and the absorbance was measured at 450 nm. Positive clones (i.e., those that reacted to *C. jejuni* but not fecal bacterial cocktail) were separated by limiting dilution.

### Preparation of monoclonal antibody

mAb was prepared as previously described (Takigawa et al., 2017). In brief, female nude mice (age, 7 weeks) were purchased from Japan SLC, Inc. (Shizuoka, Japan). Hybridoma was cultured in DMEM medium (4.5 g/L glucose, Nacalai Tesque) containing 10% (vol/vol) fetal bovine serum (Gibco, Invitrogen, Waltham, MA), 50 μM 2-mercaptoethanol (Gibco), 1 mM sodium pyruvate (Nacalai Tesque), and non-essential amino acids solution (Nacalai Tesque) at 37°C in 10% CO_2_. Mice received 500 μL pristane (Sigma) and were intraperitoneally injected with hybridoma cells (10^7^ cells/mouse) 7 days later. Then, at 10–14 days after the injection, the ascites was collected and mAbs were purified by using a protein G column (GE HealthCare). The eluted antibodies were loaded onto a PD-10 column (GE HealthCare) for exchange into PBS. The concentration of the purified antibody was determined by measuring the absorbance at 280 nm.

### Flow cytometric analysis for bacterial cells

Bacterial strains listed in Supplementary Table S3 were cultured slightly aerobically at 37°C for 2 days on Columbia 5% Horse Blood agar plates, and the bacterial cells were harvested and suspended in PBS (2 × 10^8^ CFU/mL). The bacterial cell suspension (50 μL) was mixed 1:1 with 1 mg/mL 2B1 mAb (50 μL in PBS) and the mixture (100 μL) was incubated for 30 min at 37°C. The cells were washed 3 times with PBS and incubated with APC-labeled anti-mouse IgG (clone Poly4053; BioLegend, San Diego, California, United States) for 30 min at 4°C. The stained bacterial cells were washed 3 times with PBS and fixed with 4% paraformaldehyde.

The stained samples were analyzed by using a MACSQuant analyzer (Miltenyi Biotech, Bergish Gladbach, Germany) and data analysis was performed by using the FlowJo 9.9 software (Tree Star, Ashland, Oregon, United States).

### Flow cytometric analysis for immune cells

For preparation of immune cells, flow cytometry was performed as described previously, with some modifications (Nagatake et al., 2014, 2019; Hosomi et al., 2020). Small intestine and colon were opened longitudinally and washed vigorously in PBS on ice. The intestinal samples were cut into ~2 cm sections and incubated in 2 (for small intestine) or 4 (for colon) mg/mL collagenase (Wako) in RPMI1640 medium (Sigma-Aldrich) containing 2% (vol/vol) newborn calf serum (Equitech-Bio, Kerrville, Texas, United States) for 15 min at 37°C with stirring. The cell suspensions were filtered through cell strainers (pore size, 100 μm; BD Biosciences, Franklin Lakes, New Jersey, United States). This treatment with collagenase was repeated once to prepare cell samples.

For flow cytometric analysis, intestinal lymphocytes were obtained from the cell samples by purification with Percoll (GE Healthcare) as a cell layer located between 40 and 75% solutions after gradient centrifugation (820 × g, 20 min, 20°C). These lymphocyte samples were stained with an anti-CD16/32 monoclonal antibody (TruStain fcX; BioLegend) and 7-AAD (BioLegend) to avoid non-specific staining and detect dead cells, respectively. The cells were further stained with the fluorescently labeled antibodies BV421-anti-CD45 (BioLegend, clone 30-F11), FITC-anti-Ly6G (BioLegend, clone 1A8), and APC-Cy7-anti-CD11b (BioLegend, clone M1/70).

The stained samples were analyzed by using a MACSQuant analyzer (Miltenyi Biotech, Bergish Gladbach, Germany) and data analysis was performed by using the FlowJo 9.9 software (Tree Star, Ashland, Oregon, United States).

### Super-resolution microscopy analysis

*Campylobacter jejuni* was cultured slightly aerobically at 37°C for 2 days on Columbia 5% Horse Blood agar plates and was collected by centrifugation at 10,000 × g for 5 min at room temperature (25°C). The bacterial cells (10^8^ CFU) were incubated with 0.5 mg/mL 2B1 mAb diluted with PBS for 30 min at 37°C. The cells were washed 3 times with PBS and were incubated with APC-labeled anti-mouse IgG (BioLegend; clone Poly4053) for 30 min at 4°C and then stained with DAPI (AAT Bioquest) and FM4-64 (Thermo Fisher Scientific). The stained bacterial cells were washed 3 times with PBS and fixed with 4% paraformaldehyde. The stained samples were analyzed by super-resolution microscopy by using an Elyra system (Zeiss).

### Immunoprecipitation and proteome analysis

*Campylobacter jejuni* strain 81–176 was cultured slightly aerobically at 37°C for 2 days on Columbia 5% Horse Blood agar plates and then collected by centrifugation at 10,000 × g for 5 min at room temperature (25°C). The bacterial cells were suspended in B-PER bacterial protein extraction reagent (Thermo Fisher Scientific) containing 1 mM EDTA at the concentration of 20 mg/mL (~200 μL) and incubated for 15 min at room temperature (25°C). They were then centrifuged at 16,000 × *g* for 20 min at room temperature (25°C), and the supernatant was mixed with Protein G Sepharose 4 Fast Flow (GE Healthcare) in the absence (mock) or presence of 2B1 mAb and incubated on a rotary shaker for 1 h at 4°C. The suspension was centrifuged at 12,000 × g for 20 min at room temperature (25°C), and electrophoresis of the pellet was performed by using a NuPAGE electrophoresis system (Life Technologies) with a 4–12% discontinuous Bis-Tris gel and MES buffer. SeeBlue Plus2 pre-stained standard (Thermo Fisher Scientific) was used as the molecular weight size marker. Total protein was stained with a Pierce Silver Stain kit (Thermo Fisher Scientific). The gel band was cut out and subjected to in-gel tryptic digestion, essentially as described (Adachi et al., 2006). Briefly, the gel pieces were destained and washed, and, after dithiothreitol reduction and iodoacetamide alkylation, the proteins were digested with porcine trypsin (modified sequencing grade; Promega, Madison, Wisconsin, United States) overnight at 37°C. The resulting tryptic peptides were extracted from the gel pieces consecutively with 30% acetonitrile containing 0.3% trifluoroacetic acid, and 100% acetonitrile. The extracts were evaporated in a vacuum centrifuge to remove organic solvent, then desalted and concentrated on reversed-phase C18 StageTips (Rappsilber et al., 2003).

Liquid chromatography-tandem mass spectrometry was performed with an UltiMate 3000 Nano LC system (Thermo Scientific, Bremen, Germany) and an HTC-PAL autosampler (CTC Analytics, Zwingen, Switzerland) coupled to a Q Exactive mass spectrometer (Thermo Scientific). Forty-five-minute gradients from 5–30% Buffer B were used. The Q Exactive instrument was operated as previously described (Adachi et al., 2022). Raw mass spectrometry data were processed with the Proteome Discoverer v2.1 software package (Thermo Fisher Scientific) by using the Sequest HT search engine and a UniProt *Campylobacter jejuni* subsp. *Jejuni* serotype O:23/36 (strain 81–176). The following parameter settings were used in the search: fully tryptic peptides with a minimum of 7 amino acids, maximum 2 missed tryptic cleavage sites, tolerances of 10 ppm for precursor ions and 0.01 Da for fragment ions, all b/y/a ions considered in the search, and dynamic modifications allowed on the N-terminus (42.0106 Da/acetyl) and methionine (15.9949 Da/oxidation). For PSM validation, a forward/reverse concatenated database was used. Only first-rank peptides were counted, and only for top-scoring proteins. The probability thresholds for all peptide/protein level FDRs were set at confidence thresholds of 0.05 (relaxed)/0.01 (stringent).

### Preparation of recombinant protein

Recombinant protein was prepared as previously described (Hosomi et al., 2019). In brief, QcrC fragment (NCBI Reference Sequence: NC_008787.1, locus tag: CJJ81176_1199) and its truncated forms were cloned into pCold I (Takara Bio Inc., Shiga, Japan) at KpnI and XhoI cloning sites by using the GenPlus cloning service (GenScript, Tokyo, Japan) (Supplementary Table S4). To obtain recombinant His-tagged proteins, the pCold I vector was transfected into *Escherichia coli* strain BL21 containing pG-Tf2 (Takara Bio Inc.) and protein production was induced in accordance with the manufacturer’s instructions. The culture pellet was sonicated in buffer A (10 mM Tris-HCl pH 8.0, 400 mM NaCl, 5 mM MgCl_2_, 0.1 mM phenylmethylsulfonyl fluoride, 1 mM 2-mercaptoethanol and 10% glycerol) and the recombinant protein was purified by using an AKTA Prime Plus chromatography system (GE HealthCare, Pittsburgh, Pennsylvania, United States) with a HiTrap HP column (GE HealthCare). The purified protein was loaded onto a PD-10 column (GE HealthCare) for exchange into PBS and its concentration was measured by using a BCA protein assay kit (Life Technologies, Carlsbad, California, United States). The purity of the protein was confirmed by using a NuPAGE electrophoresis system (Life Technologies) followed by staining with Coomassie Brilliant Blue.

### Western blot analysis

*Campylobacter jejuni* strain 81–176 was cultured slightly aerobically at 37°C for 2 days on Columbia 5% Horse Blood agar or Bolton agar plates or in Bolton broth. *Escherichia coli* that expressed recombinant proteins was cultured in accordance with the manufacturer’s instructions. The bacterial cells were collected by centrifugation at 10,000 × g for 5 min at room temperature (25°C). Electrophoresis of the bacterial pellet (50 μg) or purified recombinant protein (2 μg) was performed by using a NuPAGE electrophoresis system (Life Technologies) with a 4–12% discontinuous Bis-Tris gel and MES buffer. SeeBlue Plus2 pre-stained standard (Thermo Fisher Scientific) was used as the molecular weight size marker. Separated proteins were transferred to polyvinylidene difluoride membranes (EMD Millipore Corporation). The antibodies used were 2B1 mAb (50 ng/mL), anti-His Tag antibody (Biolegend) (100 ng/mL) that verified the protein expression, and horseradish-peroxidase-conjugated anti-mouse IgG (Southern Biotech). Images were captured using an ImageQuant LAS 4000 instrument (Fujifilm) and quantitative data analysis was performed using Multi Gayge v3.1 Imaging Software (Fujifilm) to calculate quantum level (QL) value.

### Flux analysis

*Campylobacter jejuni* strain 81–176 was cultured slightly aerobically at 37°C for 2 days on Columbia 5% Horse Blood agar or Bolton agar plates or in Bolton broth. The bacterial cells were collected by centrifugation at 10,000 × g for 5 min at room temperature (25°C), suspended in PBS at 5.0 × 10^7^ CFU/mL in the absence or presence of 0.5 mg/mL 2B1 mAb or 50% sera collected from mice. After incubation for 1 h at 37°C, 100 μL of the cell suspension was seeded in a 15 μg/mL Cell-Tak-coated Seahorse 24-well plate (Corning), the plate was centrifuged at 1,400 × g for 10 min at room temperature (25°C), and then 400 μL of Brucella broth (BD Biosciences) pre-warmed to 37°C was added. After 1 h pre-equilibration, the OCR was measured by using a Seahorse Bioscience XF24 Extracellular Flux Analyzer (Agilent Technologies). Xfe Wave software (Agilent Technologies) was used to analyze the results.

### Growth study of *Campylobacter jejuni*

*Campylobacter jejuni* strain 81–176 was cultured slightly aerobically at 37°C for 2 days on Columbia 5% Horse Blood agar or in Bolton broth. The bacterial cells were collected by centrifugation at 10,000 × g for 5 min at room temperature (25°C), suspended in PBS at 1–3 × 10^3^ CFU/mL in the absence or presence of 0.5 mg/mL 2B1 mAb or 50% sera collected from mice. After incubation for 1 h at 37°C, 100 μL of the cell suspension was pated on Columbia 5% Horse Blood agar and the number of *C. jejuni* colonies was counted.

### Infection model

Male BALB/c mice (age, 7 weeks) were purchased from CLEA Japan, Inc. (Tokyo, Japan). Mice were supplied with drinking water containing 0.5 g/L vancomycin (Wako) for 3 days and normal water (without vancomycin) for 1 day, then *C. jejuni* strain 81–176 (10^6^ CFU), which had been pre-treated with 2B1 mAb (1 mg/mL in PBS) for 1 h at 37°C or not, suspended in 500 μL of Brucella broth was orally administered to the mice. Fecal samples were collected and the number of *C. jejuni* colonies was counted by using *Campylobacter* blood-free selective agar base (Oxoid) supplemented with CCDA supplement (Oxoid).

### MPO ELISA

Fecal samples were collected, immediately frozen in liquid nitrogen, and stored at −80°C. Samples were suspended at 100 mg/mL in 1 mM Tris (pH 7.5), 200 mM NaCl, 5 mM EDTA buffer containing 1% protease inhibitor cocktail (Sigma-Aldrich) and PhosStop phosphatase inhibitor (1 tablet/10 mL) (Roche), mixed by vortexing for 10 min and centrifuged at 1500 × g for 15 min at 4°C. MPO was measured in the supernatant by using an MPO ELISA kit (Hycult Biotech Inc., PA, United States).

### Histologic analysis

Frozen colon tissue was analyzed histologically as described previously with some modifications (Nagatake et al., 2018). Tissue samples were washed with PBS on ice and frozen in Tissue-TeK OCT compound (Sakura Finetek, Tokyo, Japan) in liquid nitrogen. Frozen tissue sections (6 μm) were prepared by using a cryostat (model CM3050 S) and were fixed for 30 min at 4°C in prechilled 95% ethanol (Nacalai Tesque), followed by 1 min at room temperature (25°C) in prechilled 100% acetone (Nacalai Tesque).

For hematoxylin and eosin staining, tissue sections were washed with running water for 10 min, stained with Mayer hematoxylin solution (Wako) for 10 min, and washed with running water for 30 min. Tissue sections were then stained with 1% eosin Y solution (Wako) for 1 min, washed with running water for 10 s, and dehydrated through increasing concentrations of ethanol (Nacalai Tesque) for 1 min at each concentration, 70 to 100%. Tissue sections underwent final dehydration in xylene (Nacalai Tesque) for 3 min and were mounted in Permount (Falma, Tokyo, Japan).

For immune-histological analysis, tissue sections were washed with PBS for 10 min and then blocked in 2% (vol/vol) newborn calf serum in PBS for 30 min at room temperature (25°C) in an incubation chamber (Cosmo Bio, Tokyo, Japan). Tissue sections were incubated with AF647-labeled anti-Ly6G monoclonal antibody (BioLegend, clone 1A8; 1:100) and PE-labeled anti-EpCAM monoclonal antibody (BioLegend, clone G8.8; 1:100) in 2% (vol/vol) newborn calf serum in PBS for 16 h at 4°C; washed once for 5 min each with 0.1% (vol/vol) Tween-20 (Nacalai Tesque) in PBS and with PBS only; and then stained with DAPI (AAT Bioquest, Sunnyvale, California, USA; 1 μM) for 10 min at room temperature (25°C) in the incubation chamber. Finally, tissue sections were washed twice with PBS, mounted in Fluoromount (Diagnostic BioSystems, Pleasanton, California, United States), and examined under a fluorescence microscope (model BZ-9000; Keyence, Osaka, Japan).

### Whole-transcriptome RNA-Seq

*Campylobacter jejuni* strain 81–176 was cultured slightly aerobically at 37°C for 2 days on Columbia 5% Horse Blood agar plates or in Bolton broth and the bacterial cells were collected by centrifugation at 10,000 × g for 5 min at 4°C. Total RNA was isolated from the cells using NucleoSpin RNA kit (Takara Bio Inc.). RNA samples were sent to Takara Bio Inc. for RNA-Seq analysis. Briefly, RNA was quantified using a NanoDrop spectrophotometer (Thermo Fisher Scientific), and the fragment size distribution of RNA was assessed by using an Agilent 2,200 TapeStation system (Agilent Technologies, Santa Clara, California, United States). Ribosomal RNA was depleted by using a Ribo-Zero Magnetic kit for gram-negative bacteria (Illumina), and a cDNA library was constructed by using an Agilent XT-Auto system (Agilent Technologies) with TruSeq Stranded mRNA Library Prep (Illumina) and IDT for Illumina -TruSeq RNA UD Indexes (Illumina) in accordance with Ribo-Zero rRNA Removal kit reference guide v02 and TruSeq Standard mRNA reference guide v00. We then performed 150-bp paired-end sequencing by using a NovaSeq 6000 whole-genome sequencing system (Illumina) with a NovaSeq 6000 S4 Reagent kit and a NovaSeq Xp 4-Line kit in accordance with the NovaSeq 6000 sequencing system guide v11 and the guide for bcl2fastq2 conversion software v2.20, dated February 2019. RNA-Seq data were analyzed using the STAR and Genedata Profiler Genome software (Genedata) to map the RNA-Seq reads to the *C. jejuni* strain 81–176 reference genome (GenBank accession No. CP000538.1) and to compute the gene expression level (as a transcript per million value) per genomic element. Gene ontology analysis was performed using TargetMine (Chen et al., 2019).

### Immunization and ELISA

Male BALB/c mice (age, 8 weeks) were subcutaneously immunized with PBS (mock as a vehicle control), QcrC (50 μg/dose), QcrC 91–208 aa (50 μg/dose), or QcrC 209–374 aa (50 μg/dose) together with Sigma Adjuvant System (Sigma) once weekly for 3 consecutive weeks. Serum was collected 1 week after the final immunization. ELISA was performed as for hybridoma screening: 96-well immunoplates (Thermo Fisher Scientific) were coated with 100 μL of lyophilized heat-killed *C. jejuni* strain 81–176 (1 mg/mL) diluted with PBS at 4°C overnight and then blocked with 1% (w/v) bovine serum albumin in PBS. Serial dilutions of serum were added to the wells and the plates were incubated for 2 h at room temperature (25°C). The plates were incubated with goat anti-mouse IgG conjugated with horseradish peroxidase (Southern Biotech) and tetramethylbenzidine peroxidase substrate, and the absorbance was measured at 450 nm. The titer was defined as the serum dilution for which the optical density was more than 0.1.

### AlphaFold structure prediction tool

Three-dimensional structure of a protein was predicted by using an open-source software, ColabFold v1.5.5: AlphaFold2 using MMseqs2.^1^ In accordance with their guidance (Mirdita et al., 2022), amino acid sequence was submitted as a query sequence and the three-dimensional structure was predicted and displayed using default parameters.

### Statistical analysis

Statistical significance was evaluated by one-way ANOVA for comparison of multiple groups and the Mann–Whitney *U*-test for two groups by using Prism 7 (GraphPad Software, La Jolla, California, USA). A *p* value less than 0.05 was considered to be significant.

## Results

### Establishment of a *Campylobacter jejuni*-specific monoclonal antibodies

**Figure 1:**
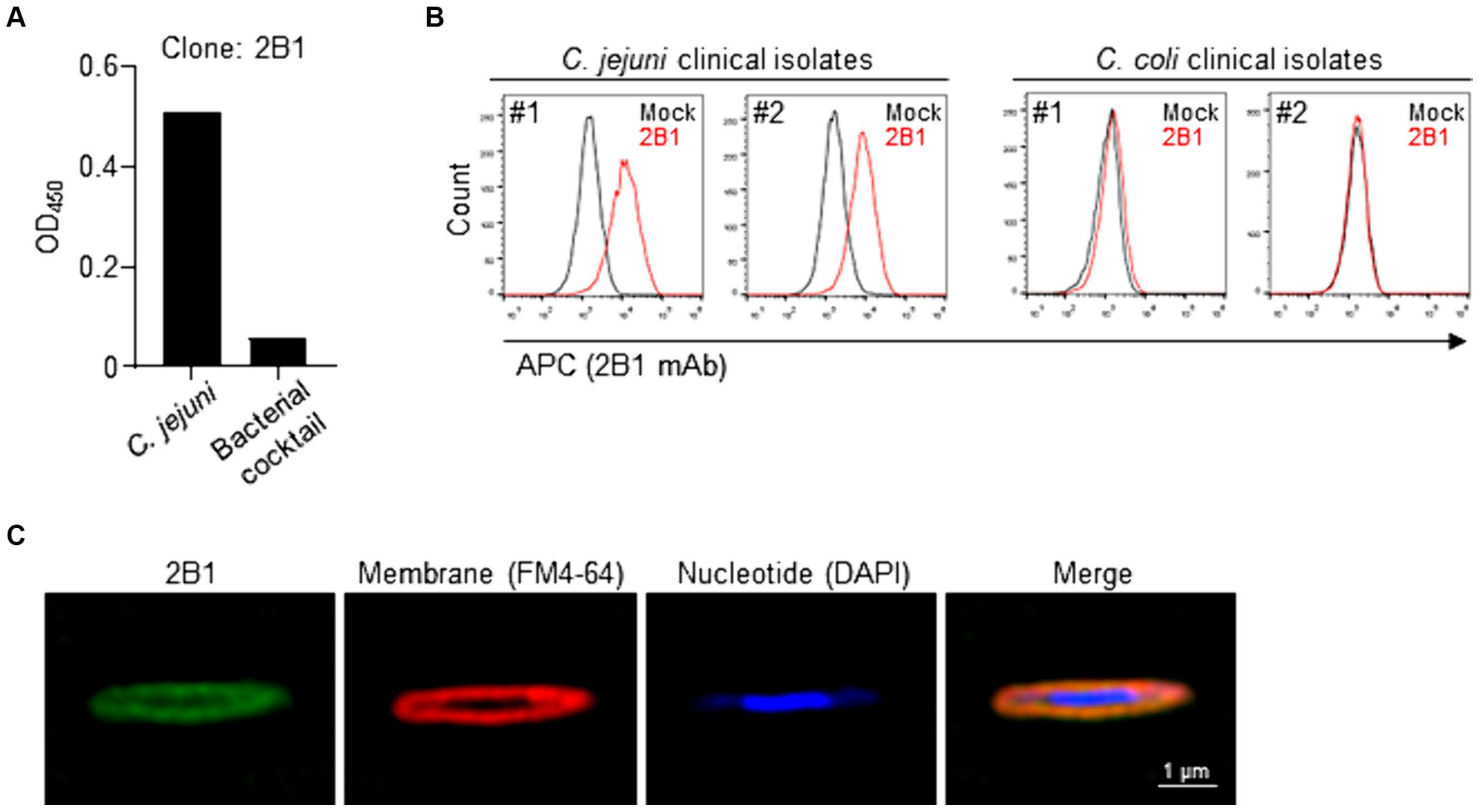
Establishment of clone 2B1, a mAb specifically reactive to *Campylobacter jejuni*. **(A)** Enzyme-linked immunosorbent assay-based screening of hybridomas that were reactive to heat-killed *C. jejuni* strain 81–176 cultured on blood agar but not to fecal bacterial cocktail. About 2,000 clones were examined and representative data for selected clone 2B1 are shown. **(B)** Reactivity of purified 2B1 to *C. jejuni* and *C. coli* clinical isolates, as determined by flow cytometry. Representative histograms are shown for *C. jejuni* or *C. coli* strains cultured on blood agar treated without (mock) or with 2B1. **(C)** Representative super-resolution microscopy images. *Campylobacter jejuni* strain 81–176 cultured on blood agar was stained with 2B1 (detected with APC-labeled anti-mouse IgG; green), FM4-64 (for cell membrane; red), and DAPI (for nucleotides; blue).

To begin to identify the functional molecules specifically expressed by *C. jejuni*, we generated a *C. jejuni*-specific monoclonal antibodies (mAbs). First, we immunized mice, one time via the footpad, with heat-killed *C. jejuni* strain 81–176 and noted that it induced swelling of the regional lymph nodes such as inguinal lymph node. Then, we established hybridomas and subjected their culture supernatants to enzyme-linked immunosorbent assay (ELISA) to select hybridomas that produced mAbs that were reactive to *C. jejuni* strain 81–176 but not to a bacterial cocktail. After this initial screening, we tested the reactivity of the obtained mAbs to clinical and environmental isolates of *C. jejuni* by flow cytometry. From this second screening, we obtained a mAb, designated clone 2B1 (mouse IgG_1_), that was reactive to all examined strains of *C. jejuni*, including several clinical strains, but not to a bacterial cocktail nor to other related species such as *C. coli* and *C. fetus*, as determined by flow cytometry (Figures 1A,B and Supplementary Table S1). Super-resolution microscopy showed that the 2B1 signal was colocalized with the FM4-64 signal for the cell membrane, but not with the DAPI signal for nucleotides (Figure 1C). Thus, 2B1 was specifically reactive to a molecule expressed on the cell membrane of the various *C. jejuni* strains tested.

### Identification of the antigen recognized by 2B1 and its binding epitope

**Figure 2:**
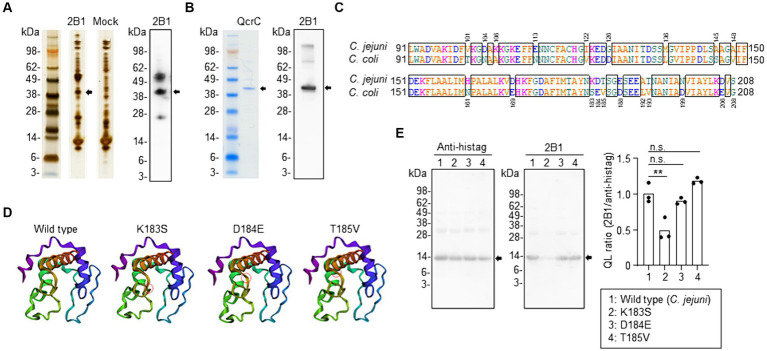
Identification of QcrC and the binding epitope of 2B1 mAb. **(A)** Immunoprecipitation of a lysate of *Campylobacter jejuni* cultured on blood agar with 2B1, and the western blot obtained with 2B1. The non-targeted bands around 50 and 25 kDa are considered to be the heavy chain and light chain, respectively, of the antibody used for immunoprecipitation. **(B)** NuPAGE electrophoresis of QcrC recombinant protein and western blot analysis using 2B1. **(C)** Comparison of amino acids 91–208 of QcrC between *C. jejuni* (A0A0H3P9E8) and *C. coli* (A0A3Z9V9Y4). **(D)** Three-dimensional structure of a *C. jejuni* QcrC fragment (amino acids 91–208) and of its three single-point mutants (K183S, D184E, T185V) predicted by using the AlphaFold structure prediction tool. Red circles indicate the location of the substituted amino acid. **(E)** Western blot analysis of the QcrC fragments shown in **(D)** by using 2B1 and anti-His-tag mAbs. Quantitative data analysis was used to calculate QL ratios (*n* = 3, mean). ^**^p < 0.01; n.s., not significant (one-way ANOVA).

To identify the molecule recognized by 2B1, we performed immunoprecipitation with *C. jejuni* lysate and 2B1; the resulting western blot showed a specific band around 40 kDa for 2B1 (Figure 2A). Proteome analysis revealed that the correlated protein was PetC (accession no. A0A0H3P9E8), an ubiquinol cytochrome *c* reductase cytochrome *c*_1_ subunit (41.5 kDa) (Supplementary Table S2), which is now designated as QcrC, a subunit of menaquinol cytochrome *c* reductase complex QcrABC (Garg et al., 2018). To confirm the specificity of 2B1 for QcrC, we produced a recombinant QcrC and confirmed that 2B1 reacted with it (Figure 2B).

To determine the binding epitope, we prepared several truncated forms of QcrC. Western blot analysis showed that 2B1 bound to the N-terminal part of QcrC (1–208 aa), not the C-terminal part (209–374 aa) (Supplementary Figure S1A). Further examinations revealed that 2B1 mAb was reactive to neither amino acids 1–198 nor 1–188 of QcrC (Supplementary Figure S1B). However, 2B1 was reactive to amino acids 71–208, 81–208, and 91–208 but not to 101–208 (Supplementary Figure S1C). Thus, 2B1 recognized QcrC via interaction with a 118-amino-acid region.

Our finding that 2B1 was reactive to *C. jejuni* but not to the related species *C. coli* (Figure 1B) prompted us to compare the amino acid sequences of the binding region (amino acids 91–208) between *C. jejuni* and *C. coli*. We found 20 individual amino acid substitutions between the two sequences (Figure 2C). We then compared the expected structures of several mutants by using the AlphaFold structure prediction tool and found that a triplet of sequential substitutions (K183S, D184E, and T185V) may influence the three-dimensional structure of the QcrC binding region (Supplementary Figure S2). Of the three substitutions, K183S and D184E appeared to induce the greatest structural changes (Figure 2D). Western blot analysis of mutants with these single-point amino acid substitutions indicated that lysine 183 of QcrC is critical for the binding of 2B1 (Figure 2E).

### Inhibition of the metabolism and growth of *Campylobacter jejuni* by 2B1

**Figure 3:**
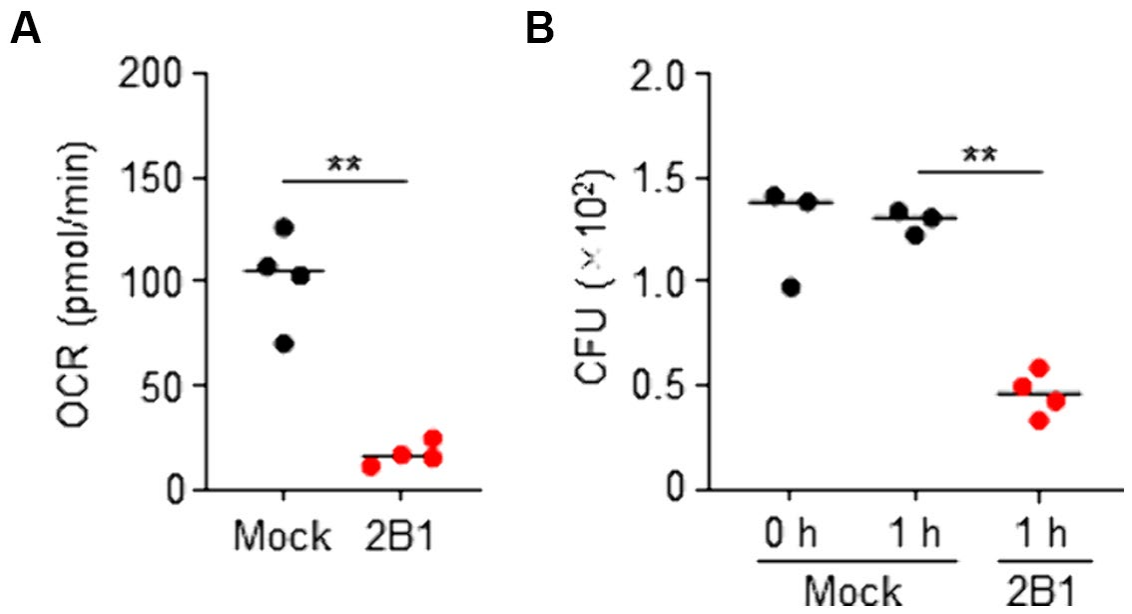
Inhibition of oxygen consumption and growth of *Campylobacter jejuni* by 2B1 mAb in microaerobic condition. **(A)** Oxygen consumption rate (OCR) of *C. jejuni* cultured slightly aerobically on blood agar and treated without (mock) or with 2B1 for 1 h at 37°C (*n* = 4, mean). Data are representative of 2 independent experiments. ^**^p < 0.01 (two-tailed Mann–Whitney *U*-test). **(B)** Colony formation of *C. jejuni* cultured slightly aerobically on blood agar and pre-treated (0 h) or treated without (mock) or with 2B1 for 1 h at 37°C (*n* = 3 or 4, mean). Data are representative of 2 independent experiments. ^**^p < 0.01 (one-way ANOVA). CFU, colony forming units.

QcrC is known to be involved in microaerobic respiration (Smith et al., 2000; Garg et al., 2018). We therefore examined the effect of 2B1 on the energy metabolism of *C. jejuni*. Flux analysis showed that treatment with 2B1 significantly reduced oxygen consumption rate (OCR) of *C. jejuni* (mean pmol/min ± SD: Mock, 102 ± 23.3; 2B1, 17 ± 5.6) (Figure 3A), suggesting inhibition of energy metabolism of *C. jejuni*. In addition, in the presence of 2B1, *in vitro* colony formation of *C. jejuni* on agar plates was significantly suppressed (mean CFU/mL ± SD: Mock, 1,292 ± 58.4; 2B1, 460 ± 107.3) (Figure 3B), suggesting inhibition of the growth of *C. jejuni*. Although it is unclear how this antibody accesses its target since QcrABC are not cell surface proteins and considered to be embedded in the inner/periplasmic membrane (Garg et al., 2018), these results suggest that 2B1 is a functional antibody that inhibits the metabolism and growth of *C. jejuni* in our culture condition.

### Relationship between QcrC expression and the metabolic activity of *Campylobacter jejuni*

**Figure 4:**
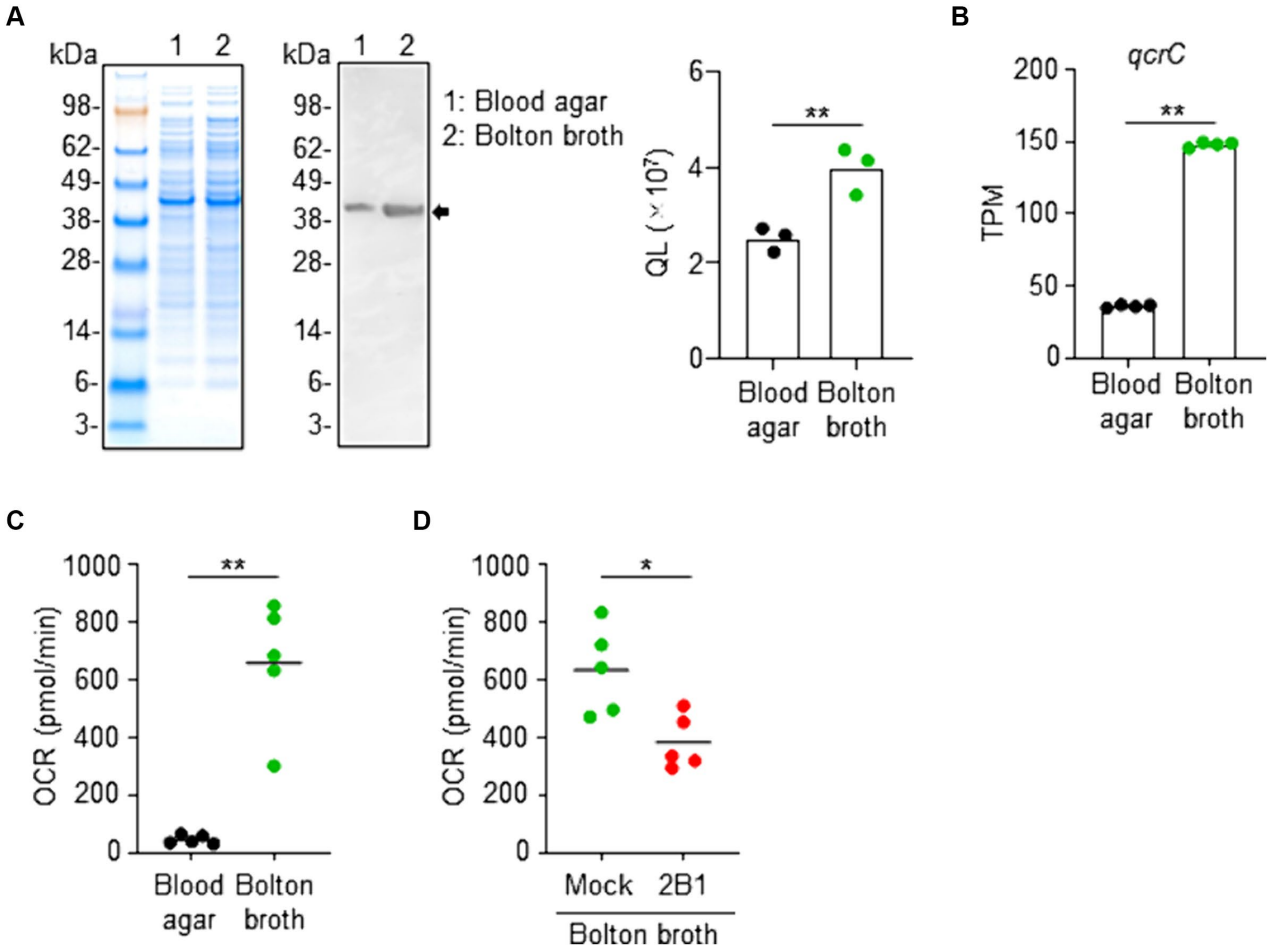
Link between QcrC expression and metabolic activity in *Campylobacter jejuni*. **(A)** NuPAGE electrophoresis of *C. jejuni* cultured slightly aerobically on blood agar or in Bolton broth, and western blot analysis using 2B1 mAb. Quantitative data analysis was used to calculate QL values (*n* = 3, mean). Data are representative of 2 independent experiments. ^**^p < 0.01 (two-tailed Mann–Whitney *U*-test). **(B)** Expression level of *qcrC* by RNAseq as transcripts per million (TPM) in *C. jejuni* cultured slightly aerobically on blood agar or in Bolton broth (*n* = 4, mean). ^**^p < 0.01 (two-tailed Mann–Whitney *U*-test). **(C)** Oxygen consumption rate (OCR) of *C. jejuni* cultured slightly aerobically on blood agar or in Bolton broth (*n* = 5, mean). Data are representative of 2 independent experiments. ^**^p < 0.01 (two-tailed Mann–Whitney *U*-test). **(D)** Oxygen consumption rate (OCR) of *C. jejuni* cultured slightly aerobically in Bolton broth and treated without (mock) or with 2B1 mAb for 1 h at 37°C (*n* = 5, mean). Data are representative of 2 independent experiments. ^*^p < 0.01 (two-tailed Mann–Whitney *U*-test).

We noted that the expression levels of QcrC differed depending on the culture conditions because *C. jejuni* is a versatile and metabolically active pathogen with a complex respiratory chains for environmental adaptation (Taylor and Kelly, 2019). Western blot analysis using 2B1 showed that QcrC expression was significantly greater in *C. jejuni* cultured in Bolton broth, a medium suitable for microaerophilic bacteria such as *Campylobacter* because it has improved aerotolerance and osmotic balance (Biesta-Peters et al., 2019), compared to that in *C. jejuni* cultured on blood agar, a widely used culture medium for *Campylobacter* (Figure 4A). RNAseq analysis supported this expression change, with the expression of *qcrC* being significantly higher in *C. jejuni* cultured in Bolton broth than in that cultured on blood agar (Figure 4B).

A gene ontology-based informatic analysis using TargetMine (Chen et al., 2019) found 3 enriched GO terms (*p* < 0.01, Benjamini–Hochberg); namely, the generation of precursor metabolites and energy (GO:0006091), carbohydrate metabolic process (GO:0005975), and developmental maturation (GO:0021700) (Supplementary Figure S3). Together with the fact that QcrC is involved in microaerobic respiration (Smith et al., 2000; Garg et al., 2018), these findings suggest metabolic changes. We next examined the metabolic state of *C. jejuni* cultured under different conditions. Flux analysis revealed that *C. jejuni* cultured in Bolton broth had a significantly increased OCR compared with *C. jejuni* cultured on blood agar (Figure 4C). Furthermore, treatment with 2B1 significantly suppressed the increased OCR of *C. jejuni* cultured in Bolton broth (Figure 4D). Together, these results indicate that QcrC expression level is linked to the metabolic activity of *C. jejuni* in our culture condition.

### Relationship between QcrC expression and pathogenicity of *Campylobacter jejuni* in mice

**Figure 5:**
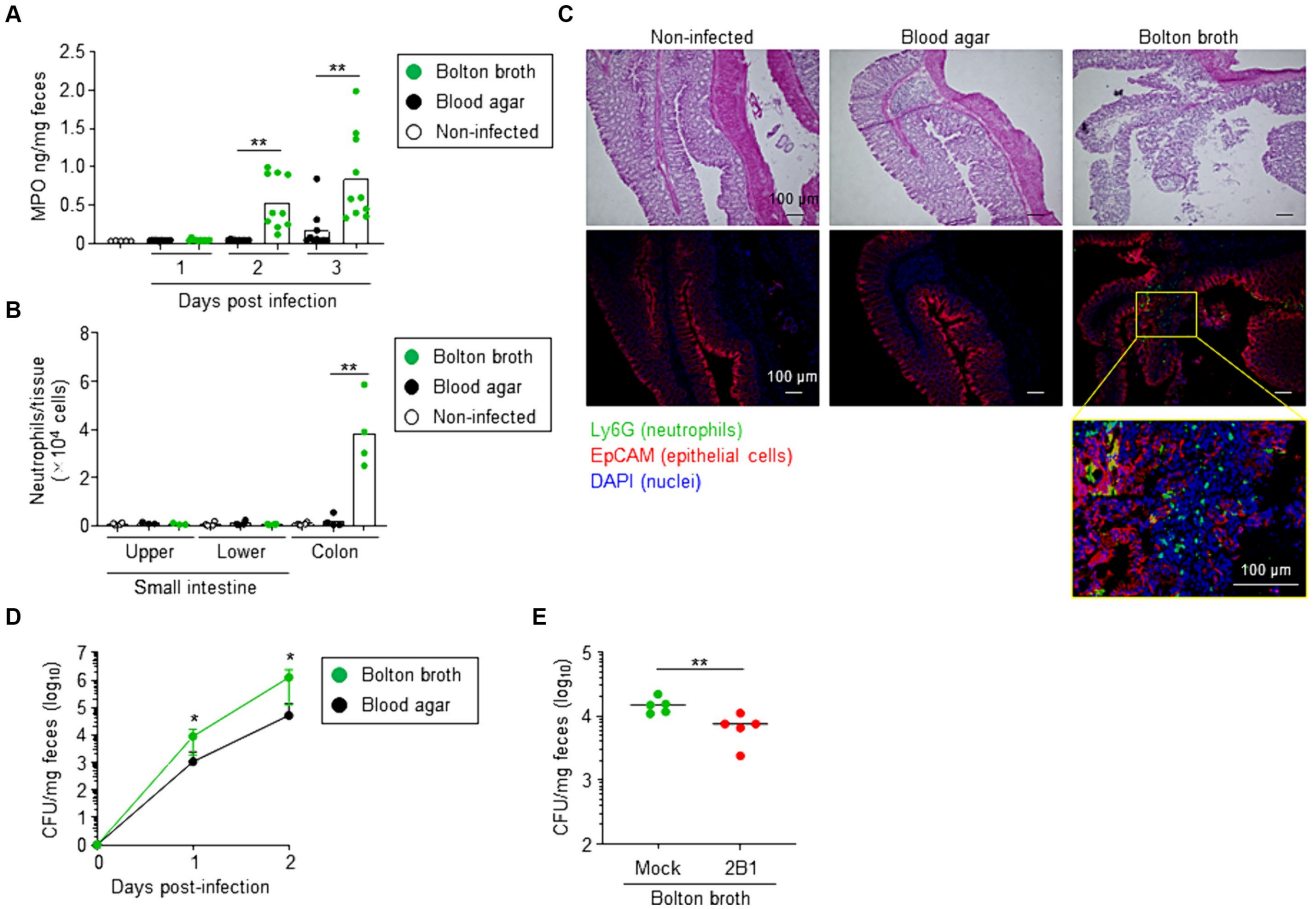
Link between QcrC expression and pathogenicity in *Campylobacter jejuni*. **(A)** Neutrophil myeloperoxidase (MPO) in feces (*n* = 10, mean). At days 1, 2, and 3 after infection, fecal samples were collected and MPO was measured. Data are combined from 2 independent experiments. ^**^p < 0.01 (two-tailed Mann–Whitney *U*-test). **(B)** Number of neutrophils in the intestine at day 3 post-infection (*n* = 4, mean). Neutrophils were defined as CD45^+^ CD11b^+^ Ly6G^high^ cells, as shown in Supplementary Figure S1, and counted in the small intestine (upper and lower sites) and colon by using flow cytometry. Data are combined from 2 independent experiments. ^**^p < 0.01 (one-way ANOVA). **(C)** Hematoxylin and eosin staining and immunohistologic analysis of colon at day 3 post-infection. Neutrophils, epithelial cells, and nuclei were visualized by using Ly6G monoclonal antibody (green), EpCAM monoclonal antibody (red), and DAPI (blue) staining, respectively. **(D)** *Campylobacter jejuni* colonization in mice (*n* = 10, mean ± 1 SD). Before infection (day 0) and on days 1 and 2 after infection with *C. jejuni* cultured in Bolton broth or on blood agar, fecal samples were collected and the number of *C. jejuni* colonies was counted by using *Campylobacter* blood-free selective agar base supplemented with CCDA supplement. Data are combined from 2 independent experiments. ^*^p < 0.05 (two-tailed Mann–Whitney *U*-test). **(E)** Intestinal colonization of *C. jejuni* cultured slightly aerobically in Bolton broth treated without (mock) or with 2B1 mAb for 1 h at 37°C (*n* = 5, mean). At day 1 after infection with *C. jejuni*, fecal samples were collected, and the number of *C. jejuni* colonies was counted by using *Campylobacter* blood-free selective agar base supplemented with CCDA supplement. Data are representative of 2 independent experiments. ^**^p < 0.01 (two-tailed Mann–Whitney *U*-test).

To assess whether increased QcrC expression is associated with the pathogenic state of *C. jejuni*, we used neutrophil myeloperoxidase (MPO) level in the feces as a marker of intestinal inflammation in a murine model of infection (de Moura Gondim Prata et al., 2016). Mice administered *C. jejuni* cultured on blood agar showed little fecal MPO throughout the experimental period, whereas fecal MPO was significantly increased on days 2 and 3 after infection in mice given *C. jejuni* cultured in Bolton broth (Figure 5A). Consistently, at 3 days post-infection, flow cytometric analysis revealed a significant increase in neutrophils in the colon of mice administered *C. jejuni* cultured in Bolton broth (Figure 5B; Supplementary Figure S4). Histological analysis further indicated neutrophil accumulation in the mucosal layer, as well as damage to the epithelial cell layer of the colon (Figure 5C).

We next examined colonization of *C. jejuni* in mice. When mice were administered *C. jejuni* cultured on blood agar, the number of fecal CFU gradually increased from day 1 to day 2 after infection (Figure 5D). Mice administered *C. jejuni* cultured in Bolton broth showed a significantly greater increase in fecal CFU (Figure 5D). However, treatment with 2B1 significantly reduced the number of intestinal bacterial colonies at day 1 post-infection compared with that in mice that did not receive 2B1 (mean CFU/mg ± SD: Mock, 15,120 ± 4,426; 2B1, 7,080 ± 3,148) (Figure 5E). These results suggest that QcrC expression level is linked to the pathogenic state of *C. jejuni* in mice.

To test this hypothesis, we next examined the culture condition in detail. One of the major differences between Bolton broth and blood agar is whether the medium state is liquid or solid. *Campylobacter jejuni* cultured on Bolton agar (solid) expressed a significantly lower level of QcrC than those cultured in Bolton broth (liquid) (Supplementary Figure S5A), and these expression levels were comparable with OCR of *C. jejuni* (Supplementary Figure S5B). Mice administered *C. jejuni* cultured on Bolton agar showed significantly fewer intestinal colonizing bacteria and significantly less fecal MPO in comparison with mice administered *C. jejuni* cultured in Bolton broth (Supplementary Figures S5C,D).

Summarizing these findings, mouse intestine was colonized most frequently by *C. jejuni* cultured in Bolton broth, and this *C. jejuni* showed the greatest expression of QcrC. In addition, mouse intestine colonized by *C. jejuni* with high QcrC expression showed the greatest intestinal inflammation.

### Potential of QcrC as a protective vaccine antigen against *Campylobacter jejuni* infection

**Figure 6:**
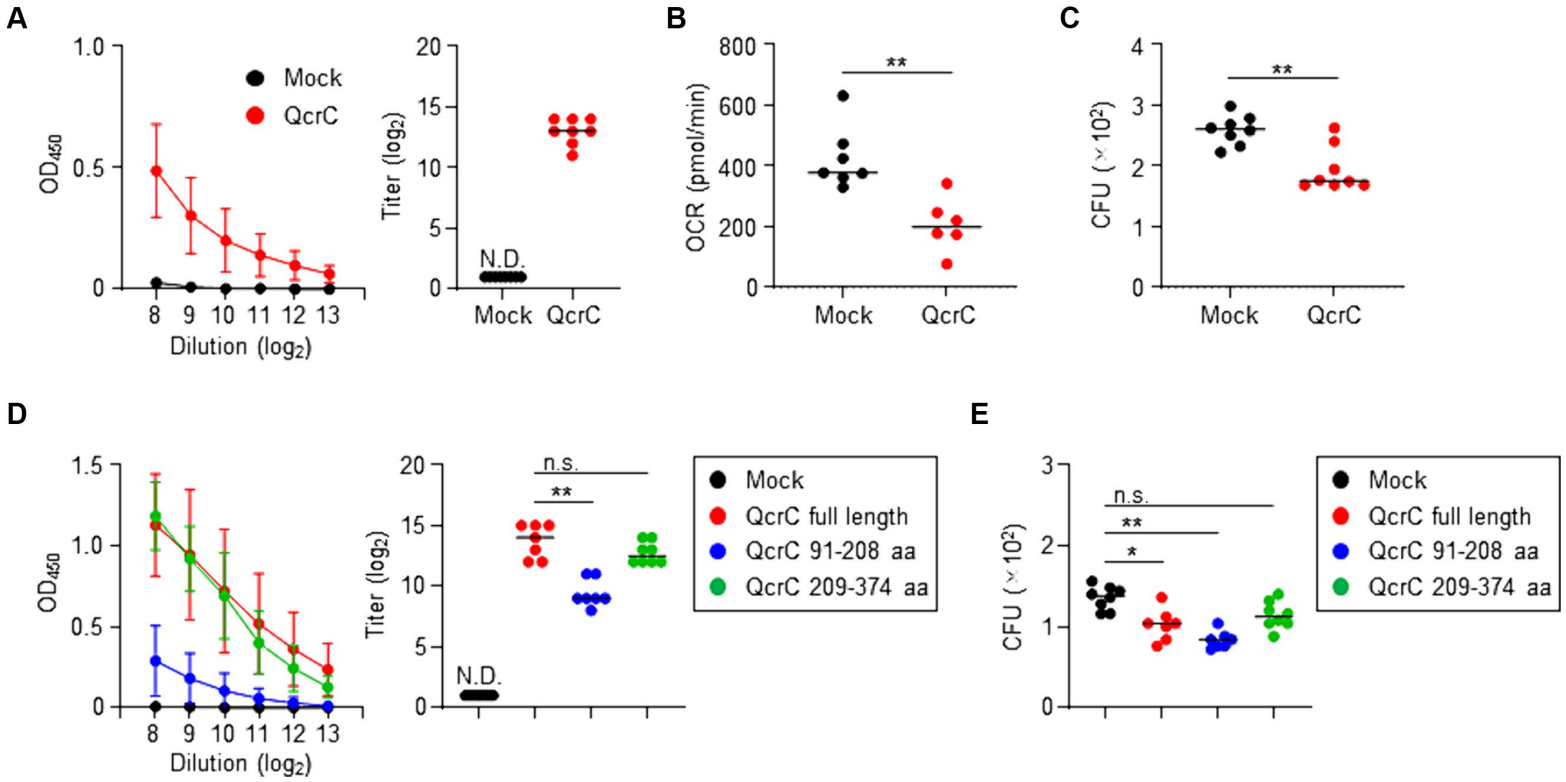
Induction of protective antibody production against *Campylobacter jejuni* by immunization with QcrC in mice. **(A)** Detection of *C. jejuni*-specific serum IgG antibody by ELISA (*n* = 8, mean ± 1 SD) in mice subcutaneously immunized with PBS (mock) or QcrC once a week for 3 consecutive weeks. The titer was defined as the serum dilution for which the optical density was more than 0.1. Data are representative of 2 independent experiments. N.D., not detected. **(B)** Oxygen consumption rate (OCR) in *C. jejuni* treated with sera collected from immunized mice (*n* = 6 or 7, mean). ^**^p < 0.01 (one-way ANOVA). **(C)** Colony formation of *C. jejuni* treated with sera collected from immunized mice (*n* = 7 or 8, mean). ^**^p < 0.01 (one-way ANOVA). **(D)** Detection of *C. jejuni*-specific serum IgG antibody by ELISA (*n* = 8, mean ± 1 SD) in mice subcutaneously immunized with PBS (mock), QcrC, QcrC 91–208 aa, or QcrC 209–374 aa once a week for 3 consecutive weeks. The titer was defined as the serum dilution for which the optical density was more than 0.1. Data are representative of 2 independent experiments. ^**^p < 0.01; n.s., not significant (one-way ANOVA); N.D., not detected. **(E)** Colony formation of *C. jejuni* treated with sera collected from immunized mice (*n* = 7 or 8, mean). Data are representative of 2 independent experiments. ^*^p < 0.05; ^**^p < 0.01; n.s., not significant (one-way ANOVA).

Because our results suggested that QcrC is associated with the pathogenicity of *C. jejuni*, we examined the potential of QcrC as a vaccine target molecule to suppress energy metabolism to control *C. jejuni* infection. Subcutaneous immunization with a recombinant QcrC in mice induced *C. jejuni*-specific serum IgG antibody production (Figure 6A). Like what was found for 2B1, treatment with sera collected from the QcrC-immunized mice significantly suppressed the OCR and growth of *C. jejuni* (Figures 6B,C). Thus, QcrC shows potential as a vaccine antigen to induce *C. jejuni*-specific neutralizing IgG antibody.

Since QcrC amino acids 91–208 are the binding epitope of 2B1 (Figure 2), we next examined the efficacy of this epitope as a vaccine antigen. Although the production level was low, subcutaneous immunization with the binding epitope induced *C. jejuni*-specific IgG antibody production in mice (Figure 6D). Notably, despite lower levels of IgG antibody production, sera collected from mice immunized with the binding epitope showed equal or greater neutralizing activity for the growth of *C. jejuni* compared with sera from mice immunized with full-length QcrC (mean CFU/mL ± SD: Mock, 1,355 ± 145.7; QcrC full length, 1,023 ± 194.4; QcrC 91–208 aa, 834.3 ± 106.9) (Figure 6E). In contrast, 2B1 mAb was not reactive to QcrC amino acids 209–374, and thus it was used as a negative control in the vaccination study of QcrC truncated forms. Immunization with QcrC amino acids 209–374 induced *C. jejuni*-specific IgG antibody production at levels comparable to immunization with full-length QcrC, but this antibody showed weak neutralizing activity (Figures 6D,E). Collectively, these results suggest that the binding epitope region of QcrC could be a promising target for the development of highly effective vaccines.

## Discussion

Intestinal environments provide a defense system against enteric pathogens (Liang and Vallance, 2021; Shealy et al., 2021). As part of this defense, commensal bacteria occupy major nutrient niches and produce inhibitory fermentation products that directly and indirectly limit pathogen expansion in the intestine (Shealy et al., 2021). To fight this system, an enteric pathogen dynamically reprograms its metabolism during infection, which allows it to overcome colonization resistance, and then expand and induce inflammation (Liang and Vallance, 2021). For example, *Citrobacter rodentium*, a murine enteric pathogen, changes its metabolism to switch from carbohydrate to amino acid utilization in the intestine so that pathogen colonization can be promoted (Caballero-Flores et al., 2020). Thus, bacterial metabolism is now considered a promising target for controlling infections.

Here, we developed a mAb, designated 2B1, that was specifically reactive to *C. jejuni*, and identified QcrC as its antigen. Notably, 2B1 could detect a wide range of *C. jejuni* strains including clinical isolates. Furthermore, the expression level of QcrC was found to be related to the metabolic activity and pathogenicity of *C. jejuni*, and the inhibitory effect of 2B1 antibody and QcrC vaccination on *C. jejuni* growth was marginal, and this is a limitation of this work. QcrC showed potential as a vaccine antigen to induce neutralizing antibody production. Despite the need for further studies on the mAb and QcrC vaccination-induced antibody that address how they work and neutralize the periplasmic target protein, we expect that these findings will contribute to the development of preventive and therapeutic approaches targeting bacterial metabolism and a diagnostic system for *C. jejuni*.

Previous studies have reported the uniqueness of the energy metabolism of *Campylobacter* and the crucial role of metabolic reprogramming in its pathogenicity. Unlike most other bacteria, *Campylobacter* lacks the ability to use carbohydrates as a carbon source for energy metabolism because it lacks the appropriate transporters to take up sugars like glucose or galactose, and it also lacks several key enzymes within the glycolytic pathway (Stahl et al., 2012; Burnham and Hendrixson, 2018). Therefore, in *C. jejuni*, amino acids such as serine are catabolized to pyruvate in the TCA cycle and utilized for bacterial growth and intestinal colonization (Hendrixson and DiRita, 2004; Velayudhan et al., 2004; Hofreuter et al., 2012), and further, metabolic differences have been shown to influence other pathogenic characteristics of *C. jejuni* such as its ability to spread between tissues (Hofreuter et al., 2008, 2012). Together, these previous studies clearly show the relationship between the metabolism and pathogenicity of *C. jejuni*. Indeed, our present findings also demonstrated that an elevated energy metabolism in *C. jejuni* is closely related to a high pathogenicity characterized by frequent colonization and severe intestinal inflammation in mice.

In the present study, we found that the pathogenic and metabolic states of *C. jejuni* are likely linked to the expression level of QcrC. *C. jejuni* is a microaerophilic bacterium and most strains grow optimally at 3–10% oxygen, with growth inhibited under either atmospheric oxygen levels or strict anaerobiosis (Taylor and Kelly, 2019). Therefore, *C. jejuni* preferentially colonizes the mucus layer and the intestinal crypts close to the epithelium, where the oxygen concentration will be higher than in the intestinal lumen (Taylor and Kelly, 2019). Qcr complex has a major role in oxygen-linked respiration in phylogenetically diverse prokaryotes (Dibrova et al., 2013). In addition to the use of oxygen as a preferred electron acceptor, *C. jejuni* can reduce nitrogen oxides that support growth under severely oxygen-limited conditions. It has been shown that nitrate and trimethylamine-N-oxide reduction and growth depend on electron transport through the Qcr complex in *C. jejuni* and considered that Qcr complex likely play role in electron transport to a wider range of oxidants than just molecular oxygen (Garg et al., 2018). Although it is unclear how 2B1 can interact with QcrC because QcrC is considered to be present in the periplasm, our results suggest that 2B1 can specifically recognize QcrC on *C. jejuni* and prevent its metabolic function, leading to the suppression of the growth of *C. jejuni*.

As to the development of a diagnostic system and vaccines, QcrC seems to be conserved among *C. jejuni* strains including clinical and environmental isolates, because it is a crucial molecule for *C. jejuni* to grow and survive via adaption to environmental conditions (Hofreuter, 2014). Therefore, although it will be necessary to pay attention to changes in expression levels due to differences in the environmental conditions, QcrC could be useful as a target for diagnosing *C. jejuni* infection owing to its high conservation across strains. Since these properties are also necessary for a vaccine antigen, in addition to induction of neutralizing immunity with QcrC vaccination, our present findings provide important insights for developing a diagnostic system and preventive/therapeutic approaches targeting bacterial metabolic components such as QcrC.

The reason why the metabolic activity of *C. jejuni* changed in different culture conditions, particularly why it decreased when *C. jejuni* was grown on agar, remains as a future research question. One possible reason could be spatial changes in the metabolic profile of *C. jejuni* colonies grown on agar. A three-dimensional agent-based model that describes the establishment of simple bacterial colonies expanding by the physical force of their growth demonstrated that radial colony expansion is limited by mechanical forces (Warren et al., 2019). Nutrient penetration instead governs vertical colony growth through thin layers of vertically oriented cells lifting up their ancestors from below (Warren et al., 2019). We speculate that the nutritional environment around a bacterial cell strongly depends on the medium state, broth (liquid) or agar (solid), even when both states contain the same components, with a subsequent change in the metabolic function of the bacterial cell. This speculation is supported by a previous morphological observation that cells with characteristic morphological forms predominate at different locations within a single colony of *C. jejuni*: actively growing spiral forms predominate at the edge, while inactive coccoid forms predominate in the center (Ng et al., 1985). Thus, bacterial colonies on agar plates have asynchronous growth and metabolic states, unlike cells growing exponentially in a liquid culture.

Several murine models for *Campylobacter* enteropathy have been proposed (Mansfield et al., 2007; Stahl et al., 2014; Giallourou et al., 2018), but they do not recapitulate natural host conditions because they require genetic and environmental manipulations of the mice. Therefore, for the development of vaccines and therapeutics against *C. jejuni*, a novel murine model that evaluates *C. jejuni* enteropathy is required to understand more about the pathogenesis of *C. jejuni* infection (Poly et al., 2019; Seo et al., 2020). In this regard, the strategy of modifying the bacterial culture condition may be useful in that it can allow to induce intestinal inflammation as a host natural condition without genetic and environmental manipulations of mice. Fecal MPO, measured as an intestinal inflammation marker in the present study, is a commonly used biomarker in both human and animal model studies (Amour et al., 2016; de Moura Gondim Prata et al., 2016). An added advantage is that the dose of *C. jejuni* needed in this model is lower than that in other models (Poly et al., 2019). Although *C. jejuni* is evolutionarily adapted to the avian gut rather than mammalian gut, and the murine model of *Campylobacter* infection has several limitations, our findings will hopefully contribute to the development of an improved rodent evaluation method for future vaccines and therapeutics.

In summary, by using 2B1 mAb as an experimental tool, we confirmed that the metabolic function and pathogenicity of *C. jejuni* are closely linked. Notably, we found that QcrC can be a marker representing both the metabolic and pathogenic states of *C. jejuni* and is a promising target for the control of *C. jejuni* infection by regulating bacterial metabolism. Thus, our present results provide important information for the development of novel strategies for the prevention, diagnosis, and treatment of *C. jejuni* infection.

## Data availability statement

The original contributions presented in the study are included in the article/Supplementary material, further inquiries can be directed to the corresponding author.

## Ethics statement

The animal study was approved by the Animal Care and Use Committees of the National Institutes of Biomedical Innovation, Health and Nutrition and of Osaka Metropolitan University. The study was conducted in accordance with the local legislation and institutional requirements.

## Author contributions

KH: Funding acquisition, Investigation, Writing – original draft, Writing – review & editing. NH: Investigation, Writing – review & editing. AH: Investigation, Writing – review & editing. JA: Investigation, Writing – review & editing. YT: Investigation, Writing – review & editing. MF: Investigation, Writing – review & editing. KU: Investigation, Writing – review & editing. MM: Investigation, Writing – review & editing. TN: Funding acquisition, Investigation, Writing – review & editing. AS: Funding acquisition, Investigation, Writing – review & editing. SKa: Investigation, Writing – review & editing. KY: Funding acquisition, Investigation, Writing – review & editing. SKo: Funding acquisition, Investigation, Writing – review & editing. SY: Funding acquisition, Supervision, Writing – review & editing. JK: Funding acquisition, Supervision, Writing – original draft, Writing – review & editing.

## Footnotes

^1^ https://colab.research.google.com/github/sokrypton/ColabFold/blob/main/AlphaFold2.ipynb
