# Supplementary material for: QcrC is a potential target for antibody therapy and vaccination to control Campylobacter jejuni infection by suppressing its energy metabolism
Source: Front Microbiol. 2024 Jul 2;15:1415893. doi: 10.3389/fmicb.2024.1415893 (PMC11250076; doi:10.3389/fmicb.2024.1415893)
Supplement: Supplementary file 5 [file Presentation_1.PPTX]

## Slide 1
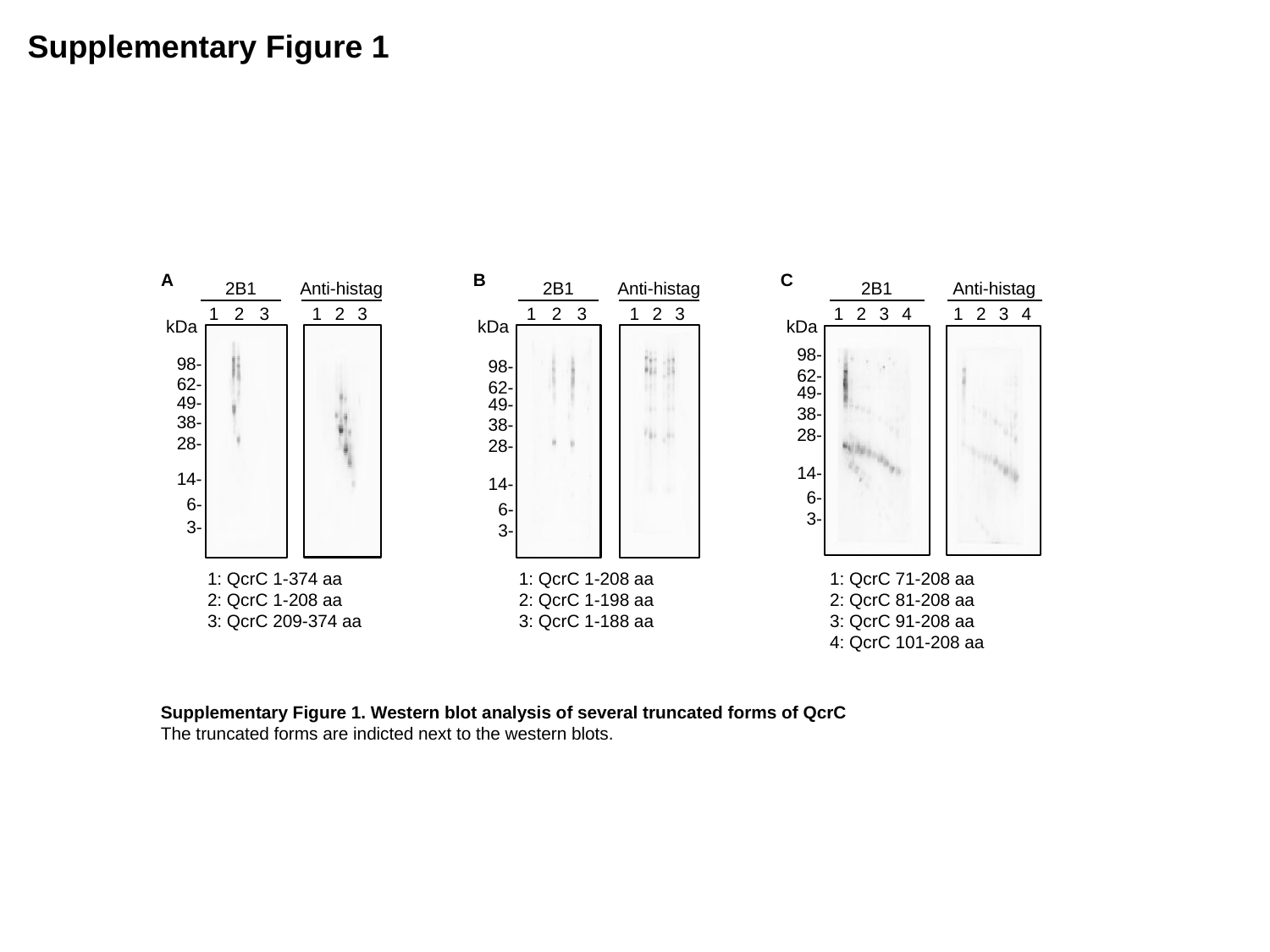

Supplementary Figure 1
A
B
C
2B1
Anti-histag
2B1
Anti-histag
2B1
Anti-histag
1
2
3
1
2
3
1
2
3
1
2
3
1
2
3
4
1
2
3
4
kDa
kDa
kDa
98-
98-
62-
49-
38-
28-
14-
6-
3-
98-
62-
62-
49-
49-
38-
38-
28-
28-
14-
14-
6-
6-
3-
3-
1: QcrC 1-374 aa
2: QcrC 1-208 aa
3: QcrC 209-374 aa
1: QcrC 1-208 aa
2: QcrC 1-198 aa
3: QcrC 1-188 aa
1: QcrC 71-208 aa
2: QcrC 81-208 aa
3: QcrC 91-208 aa
4: QcrC 101-208 aa
Supplementary Figure 1. Western blot analysis of several truncated forms of QcrC
The truncated forms are indicted next to the western blots.

## Slide 2
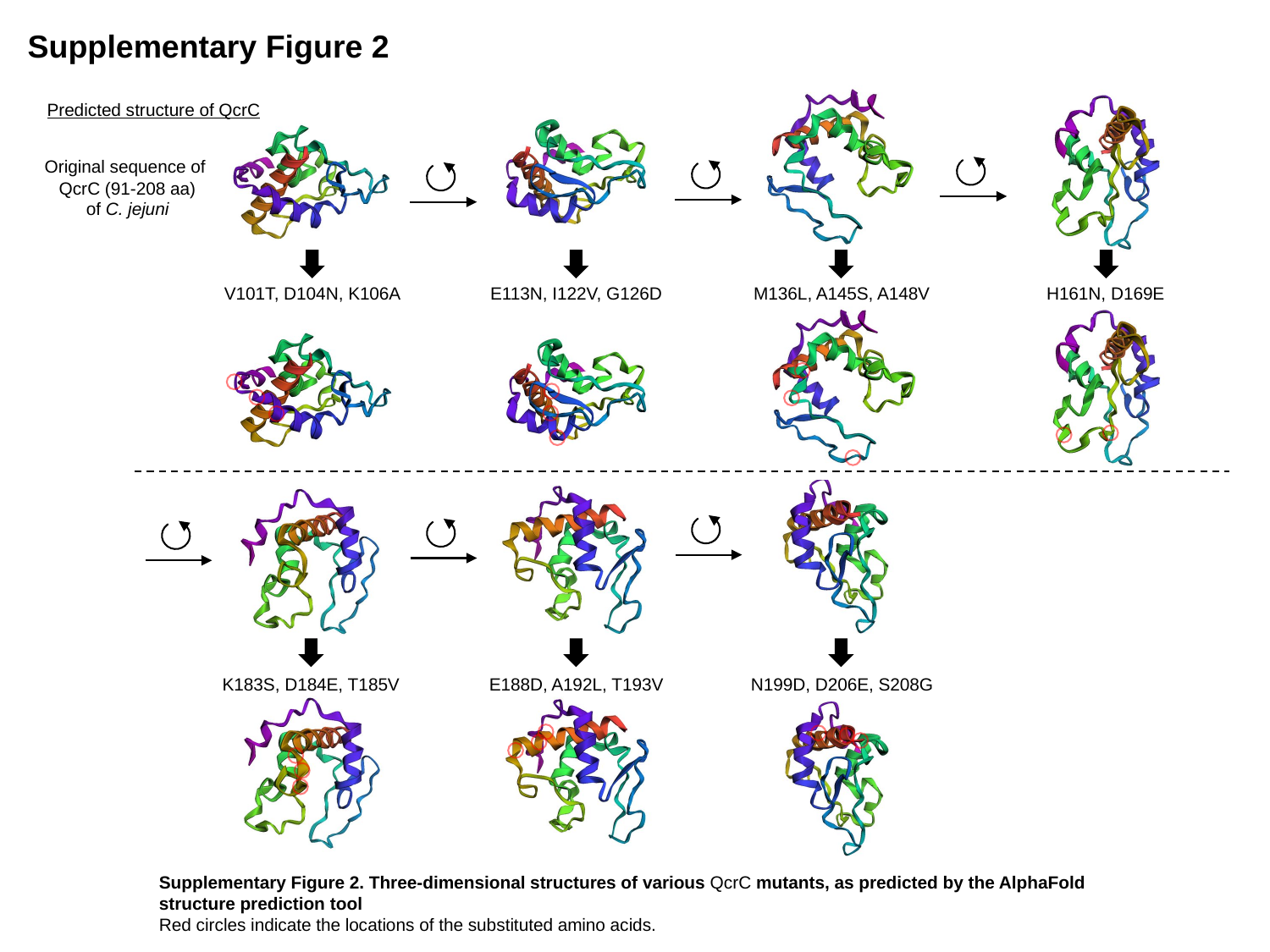

Supplementary Figure 2
Predicted structure of QcrC
Original sequence of
QcrC (91-208 aa)
of C. jejuni
V101T, D104N, K106A
E113N, I122V, G126D
M136L, A145S, A148V
H161N, D169E
K183S, D184E, T185V
E188D, A192L, T193V
N199D, D206E, S208G
Supplementary Figure 2. Three-dimensional structures of various QcrC mutants, as predicted by the AlphaFold structure prediction tool
Red circles indicate the locations of the substituted amino acids.

## Slide 3
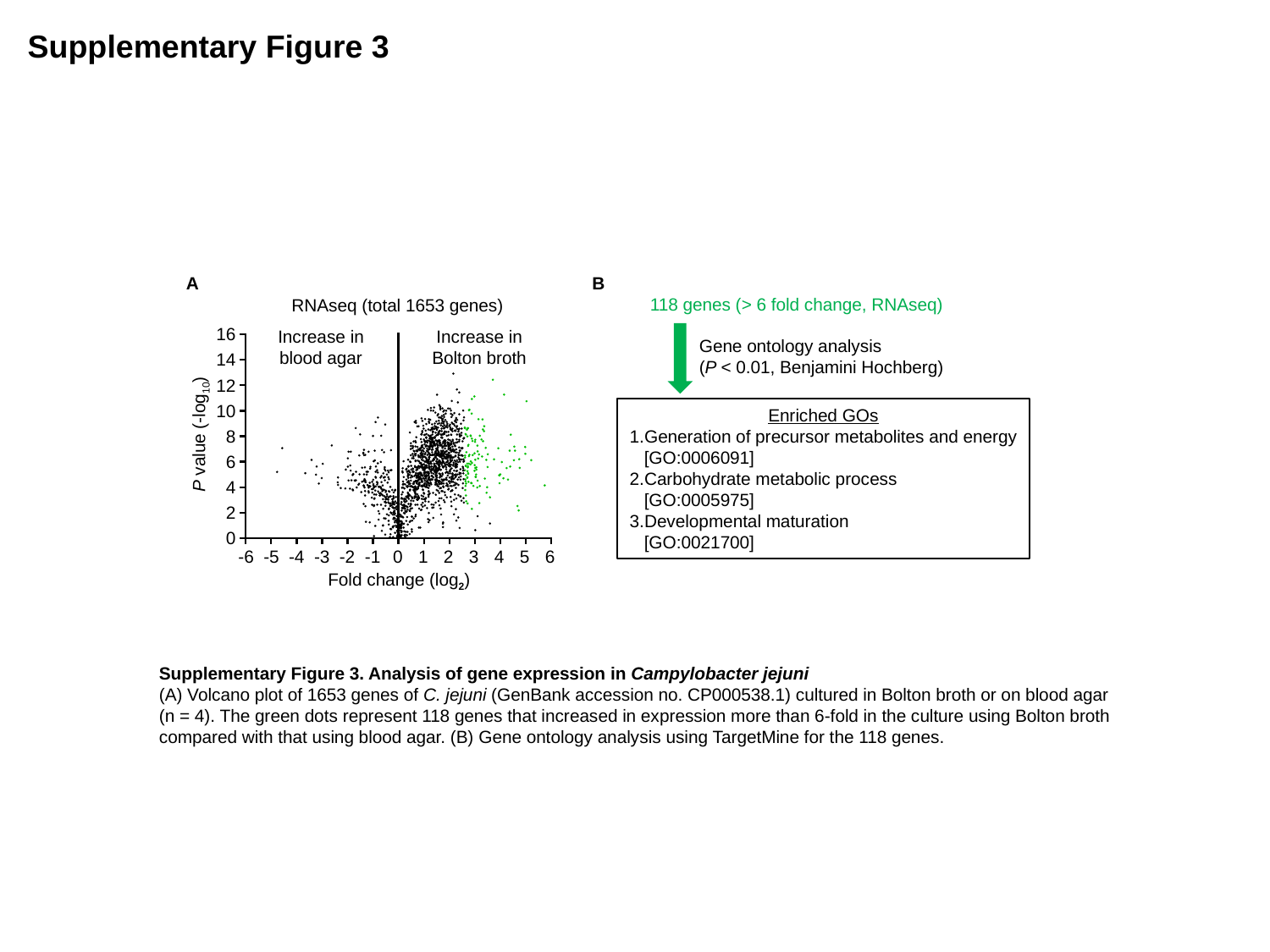

Supplementary Figure 3
A
B
118 genes (> 6 fold change, RNAseq)
Gene ontology analysis
(P < 0.01, Benjamini Hochberg)
Enriched GOs
1.Generation of precursor metabolites and energy
 [GO:0006091]
2.Carbohydrate metabolic process
 [GO:0005975]
3.Developmental maturation
 [GO:0021700]
RNAseq (total 1653 genes)
16
Increase in
blood agar
Increase in
Bolton broth
14
12
10
P value (-log10)
8
6
4
2
0
-6
-5
-4
-3
-2
-1
0
1
2
3
4
5
6
Fold change (log2)
Supplementary Figure 3. Analysis of gene expression in Campylobacter jejuni
(A) Volcano plot of 1653 genes of C. jejuni (GenBank accession no. CP000538.1) cultured in Bolton broth or on blood agar (n = 4). The green dots represent 118 genes that increased in expression more than 6-fold in the culture using Bolton broth compared with that using blood agar. (B) Gene ontology analysis using TargetMine for the 118 genes.

## Slide 4
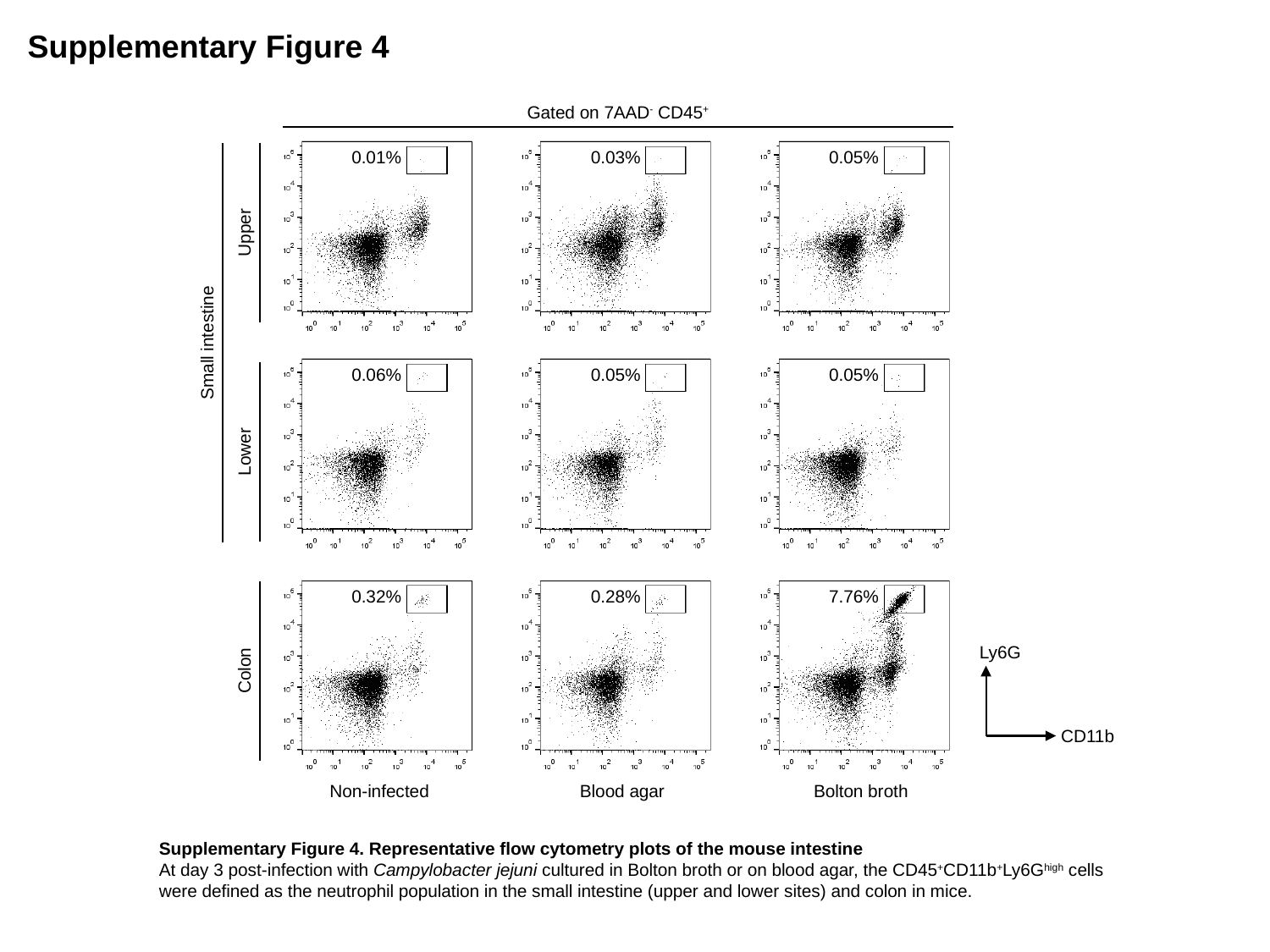

Supplementary Figure 4
Gated on 7AAD- CD45+
0.01%
0.03%
0.05%
Upper
Small intestine
0.06%
0.05%
0.05%
Lower
0.32%
0.28%
7.76%
Ly6G
CD11b
Colon
Non-infected
Blood agar
Bolton broth
Supplementary Figure 4. Representative flow cytometry plots of the mouse intestine
At day 3 post-infection with Campylobacter jejuni cultured in Bolton broth or on blood agar, the CD45+CD11b+Ly6Ghigh cells were defined as the neutrophil population in the small intestine (upper and lower sites) and colon in mice.

## Slide 5
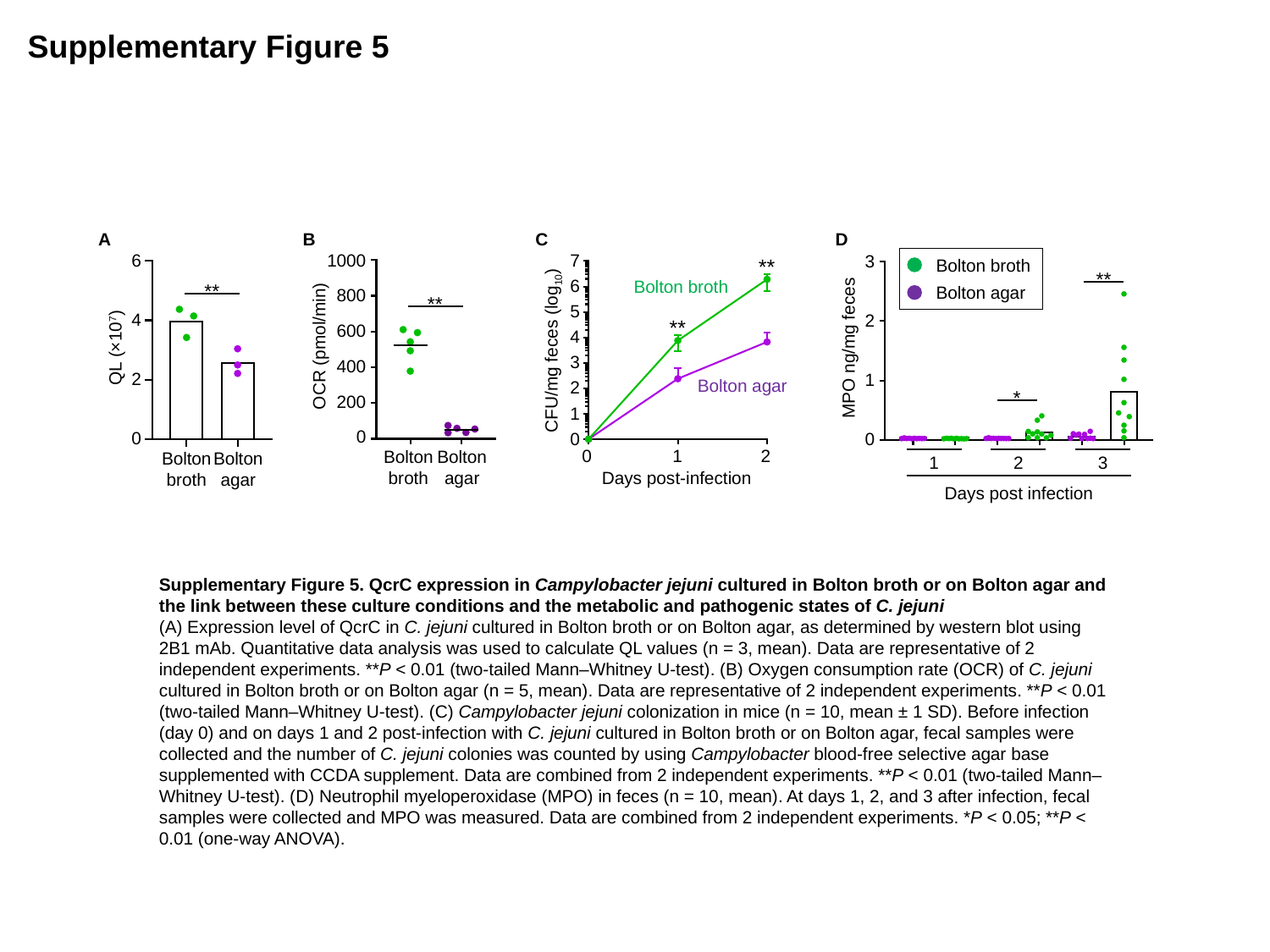

Supplementary Figure 5
A
B
C
D
1000
7
6
3
**
Bolton broth
Bolton agar
**
6
Bolton broth
**
800
**
5
4
2
**
600
4
OCR (pmol/min)
QL (×107)
MPO ng/mg feces
CFU/mg feces (log10)
3
400
2
1
Bolton agar
2
*
200
1
0
0
0
0
0
1
2
Bolton
broth
Bolton
agar
Bolton
broth
Bolton
agar
1
2
3
Days post-infection
Days post infection
Supplementary Figure 5. QcrC expression in Campylobacter jejuni cultured in Bolton broth or on Bolton agar and the link between these culture conditions and the metabolic and pathogenic states of C. jejuni
(A) Expression level of QcrC in C. jejuni cultured in Bolton broth or on Bolton agar, as determined by western blot using 2B1 mAb. Quantitative data analysis was used to calculate QL values (n = 3, mean). Data are representative of 2 independent experiments. **P < 0.01 (two-tailed Mann–Whitney U-test). (B) Oxygen consumption rate (OCR) of C. jejuni cultured in Bolton broth or on Bolton agar (n = 5, mean). Data are representative of 2 independent experiments. **P < 0.01 (two-tailed Mann–Whitney U-test). (C) Campylobacter jejuni colonization in mice (n = 10, mean ± 1 SD). Before infection (day 0) and on days 1 and 2 post-infection with C. jejuni cultured in Bolton broth or on Bolton agar, fecal samples were collected and the number of C. jejuni colonies was counted by using Campylobacter blood-free selective agar base supplemented with CCDA supplement. Data are combined from 2 independent experiments. **P < 0.01 (two-tailed Mann–Whitney U-test). (D) Neutrophil myeloperoxidase (MPO) in feces (n = 10, mean). At days 1, 2, and 3 after infection, fecal samples were collected and MPO was measured. Data are combined from 2 independent experiments. *P < 0.05; **P < 0.01 (one-way ANOVA).
